# The revolutionary role of machine learning in predicting, diagnosing, and treating liver disease

**DOI:** 10.1016/j.livres.2025.04.001

**Published:** 2025-04-14

**Authors:** Xiang-Long Huang, Wen-Hao Chen, Min Ding, Yu-Mu Song, Yun-Wen Zheng

**Affiliations:** Institute of Regenerative Medicine, and School of Medicine, Jiangsu University, Zhenjiang, Jiangsu, China; Guangdong Provincial Key Laboratory of Large Animal Models for Biomedicine, and South China Institute of Large Animal Models for Biomedicine, School of Pharmacy and Food Engineering, Wuyi University, Jiangmen, Guangdong, China; Institute of Regenerative Medicine, and School of Medicine, Jiangsu University, Zhenjiang, Jiangsu, China; Guangdong Provincial Key Laboratory of Large Animal Models for Biomedicine, and South China Institute of Large Animal Models for Biomedicine, School of Pharmacy and Food Engineering, Wuyi University, Jiangmen, Guangdong, China; Haihe Laboratory of Cell Ecosystem, Institute of Hematology, Chinese Academy of Medical Sciences, Tianjin, China; Guangdong Provincial Key Laboratory of Large Animal Models for Biomedicine, and South China Institute of Large Animal Models for Biomedicine, School of Pharmacy and Food Engineering, Wuyi University, Jiangmen, Guangdong, China; Prometheus RegMed Tech (Suzhou) Co., Ltd, Kunshan, Jiangsu, China; Department of Medicinal and Life Sciences, Faculty of Pharmaceutical Sciences, Tokyo University of Science, Tokyo, Japan; Haihe Laboratory of Cell Ecosystem, Institute of Hematology, Chinese Academy of Medical Sciences, Tianjin, China; Guangdong Provincial Key Laboratory of Large Animal Models for Biomedicine, and South China Institute of Large Animal Models for Biomedicine, School of Pharmacy and Food Engineering, Wuyi University, Jiangmen, Guangdong, China; Institute of Regenerative Medicine, and Department of Dermatology, Affiliated Hospital of Jiangsu University, Jiangsu University, Zhenjiang, Jiangsu, China

*Dear Editor,*

Liver diseases, as a significant global health issue, are characterized by complex and diverse pathogenic mechanisms. In recent years, the rapid development of biotechnology, particularly advancements in single-cell omics, genomics, and metabolomics, has led to significant improvements in the early diagnosis, treatment, and regeneration research of liver diseases.^1^ In this process, machine learning (ML) has emerged as a powerful data analysis tool that is widely applied in various fields, including research on liver diseases and regeneration.^2^, ^3^

The 2024 Nobel Prize in Physics underscores the extensive application of ML and artificial intelligence (AI) in simulated experiments and data analysis.^4^ ML, which is a subset of AI, is the science of enabling computers to simulate human learning processes by analyzing data to make predictions or decisions without being explicitly programmed based on existing knowledge. AI is a broad field of computer science dedicated to developing systems that can execute tasks that usually require human intelligence. Although AI cannot autonomously retrieve and analyze data, it relies on user-provided data or specific tools (such as web search application programming interface) to obtain information. Large-scale ML serves as an important means of achieving AI, and AI also encompasses ML and leverages it to enhance its capabilities. ML outcomes contribute to the creation of AI systems, making them more intelligent.

Conventional research directions in ML primarily include studies on decision trees, random forests, artificial neural networks, Bayesian learning, and related areas. ML has outperformed conventional medical technologies by achieving advancements in automatic visual recognition, image classification, data analysis, and trend prediction in medical image diagnosis, patient data analysis, and disease forecasting. It has emerged as an increasingly important tool in scientific research and has been successfully utilized in the early prediction, diagnosis, and treatment of liver diseases.^5^, ^6^

ML models have been employed for the prediction and early diagnosis of liver diseases, offering patients more treatment options and increasing long-term survival rates. For instance, in the diagnosis of fatty liver disease (FLD), conventional liver biopsy is not the preferred choice because of its invasiveness and low patient compliance, whereas computed tomography (CT) scans are costly, slow, and difficult to implement for large-scale screening. Weng *et al*.^7^ developed an ML model for screening individuals at high risk of FLD. Using abdominal ultrasonography for diagnosis, they established eight classification models based on 11 clinical indicators and performed a comprehensive evaluation. The results showed that the extreme gradient boosting (XGBoost) model achieved the highest prediction accuracy of 89.77%. This model can effectively improve the efficiency and cost-effectiveness of FLD screening, making it particularly suitable for large-scale screening.^7^

Before the ML era, early screening and risk assessment of liver diseases largely relied on a doctor's manual analysis of extensive clinical data, encompassing medical history, laboratory tests, and imaging studies. In particular, as regards imaging data (such as CT and magnetic resonance imaging (MRI)) and blood tests, doctors had to spend a significant amount of time integrating and analyzing data from various sources. ML can simulate human data processing by learning from and summarizing large volumes of data, thereby automating these processes through artificial neural networks. By using deep-learning models, such as convolutional neural networks (CNNs), images can be automatically analyzed, or integrated ML models can be applied to quickly process blood markers and medical records, significantly improving the efficiency of the workflow.

CNNs are a class of deep-learning models designed to process image data. They mimic the structure and function of the biological visual system, so they can automatically extract features from images and perform classification tasks. Gao *et al*.^8^ proposed a deep-learning model *Spatial Extractor-Temporal Encoder-Integration-Classifier* (STIC) based on multiphase-enhanced CT and clinical features. This model effectively extracts and integrates imaging and clinical features by combining deep CNNs and gated recurrent NNs, helping doctors accurately distinguish malignant liver tumors, thereby improving diagnostic accuracy and sensitivity. Hamm *et al*.^9^ developed and validated a CNN-based deep-learning system for diagnosing common hepatic lesions on multiphase MR images. The system demonstrated high accuracy, sensitivity, and specificity, highlighting its potential in diagnosing hepatic lesions. It confirmed the feasibility of diagnosing six prevalent hepatic lesion types based on standard imaging features, laying a foundation for future research using larger, multi-institutional datasets and more complex imaging presentations.^9^

However, ML has been relatively underutilized in the treatment of liver diseases, primarily being used to develop adjunctive treatment strategies, such as personalized treatment decisions and liver transplant management. For instance, Ji *et al*.^10^ developed an ML-based model to predict survival outcomes following liver resection in patients with early-stage hepatocellular carcinoma. The model integrates eight clinical variables (including age, race, alpha fetoprotein, tumor size, multifocality, vascular invasion, histological grade, and fibrosis score) and effectively predicts disease-specific survival (DSS), with a C-statistic of >0.72, outperforming conventional staging systems. This model enables risk stratification, with 10-year DSS rates ranging from 70% in the low-risk group to 5% in the high-risk group.^10^ With continued advancements in ML, its role in the treatment of liver diseases will become increasingly pivotal in the future.

Despite the potential of ML in liver research and treatment, the complexity of liver diseases and regeneration demands an interdisciplinary integration of multidimensional data, such as genomics, transcriptomics, proteomics, and metabolomics. More advanced ML algorithms are essential to uncover the complex mechanisms underlying liver diseases and regeneration. Furthermore, the success of ML applications in liver diseases is heavily dependent on the availability of large, high-quality datasets. To achieve its comprehensive and effective clinical applications, technical and trust-related challenges must be addressed, alongside ongoing research and optimization (Fig. 1).

**Fig. 1:**
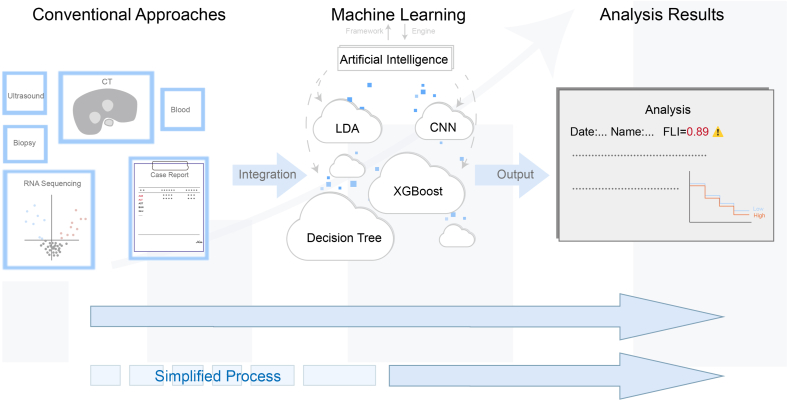
**Application of machine learning in liver diseases.** Conventional diagnostic approaches include computed tomography (CT), biochemical marker testing, ultrasonography, biopsy, and RNA sequencing. Artificial intelligence, which is the framework, integrates data using various machine learning algorithms such as LDA, CNN, etc. Machine learning algorithms automatically process and analyze large datasets to deliver efficient results, enabling earlier disease risk prediction and more effective interventions while significantly shortening the diagnostic timeline compared with conventional diagnostic approaches. Abbreviations: CNN, convolutional neural network; LDA, linear discriminant analysis; XGBoost, extreme gradient boosting.

In conclusion, ML revolutionizes liver disease management through enhanced diagnostics and personalized treatment predictions, outperforming conventional approaches in accuracy and efficiency. However, it has some limitations, such as data scarcity for rare diseases, limited model generalizability across populations, and the “black box” nature of AI systems hindering clinical trust. Priorities include federated learning for data sharing, hybrid models integrating multiomics/imaging data, and explainable AI frameworks. Emerging quantum ML and organ-on-chip technologies may facilitate predictive, individualized disease management, advancing toward precision cures. As these technical and translational challenges are systematically addressed, ML is set to evolve from being an auxiliary diagnostic tool to becoming the cornerstone of precision hepatology—potentially achieving disease interception in presymptomatic phases and personalized regenerative therapies.

## Authors’ contributions

**Yun-Wen Zheng:** Writing – review & editing, Funding acquisition, Conceptualization. **Xiang-Long Huang:** Writing – review & editing, Writing – original draft, Funding acquisition. **Wen-Hao Chen:** Writing – review & editing, Writing – original draft. **Ming Ding:** Writing – review & editing, Visualization. **Yu-Mu Song:** Writing – review & editing.

## Declaration of competing interest

The authors declare the following financial interests or personal relationships which may be considered as potential competing interest: Xiang-Long Huang reports financial support was provided by Jiangsu University. Yu-Mu Song reports a relationship with Prometheus RegMed Tech (Suzhou) Co., Ltd that includes: employment. Other authors declare that they have no known competing financial interests or personal relationships that could have appeared to influence the work reported in this paper.
